# SIRT7-mediated ACSL3 delactylation suppresses colorectal cancer progression by orchestrating tumor-associated macrophage ferroptosis and polarization

**DOI:** 10.1038/s41419-026-09007-2

**Published:** 2026-06-30

**Authors:** Lingzhen Jiang, Chaoqun Li, Peng Teng, Yan Qin, Junhui Tang, Xiaohui Hu, Yuna Li, Feiyu Qin, Surui Yao, Xiaowei Qi, Zhaohui Huang, Yuan Yin

**Affiliations:** 1 Wuxi Cancer Institute, Affiliated Hospital of Jiangnan University, Wuxi, China; 2 Laboratory of Cancer Epigenetics, Wuxi School of Medicine, Jiangnan University, Wuxi, China; 3 Department of Pathology, Affiliated Hospital of Jiangnan University, Wuxi, China

**Keywords:** Post-translational modifications, Tumour immunology

## Abstract

Dysregulated metabolism and functions of immune cells in the tumor microenvironment (TME) are key factors contributing to cancer progression. As the core immune cells in TME, tumor-associated macrophages (TAMs) have become a central focus in metabolic research of TME, though underlying mechanisms are not fully elucidated. Here, we found that long-chain acyl-coenzyme A synthetase-3 (ACSL3) is highly expressed in TAMs and correlates with poor prognosis in colorectal cancer (CRC). ACSL3 depletion induces TAM ferroptosis and reprograms them toward an M1-like phenotype. We further revealed that ACSL3 undergoes lactylation in TAMs, and identified Sirtuin 7 (SIRT7) as its delactylase. Mechanistically, SIRT7 delactylates ACSL3 at lysine 679, restores its enzymatic function and reprograms lipid metabolism in TAMs. Importantly, lactylated and activated ACSL3 significantly protects TAMs from ferroptotic stress and sustains their immunosuppressive phenotype. On the contrary, inhibiting ACSL3 lactylation reprograms TAMs toward an M1-like phenotype, further restores T cell cytotoxicity and suppresses CRC progression. Collectively, these findings establish ACSL3 lactylation in TAMs as a crucial signaling event that enables ferroptosis resistance and maintains M2 polarization, thereby promoting CRC progression. This study provides the therapeutic potential of targeting SIRT7-mediated delactylation of ACSL3 in TAMs, offering a promising strategy for CRC therapy.

## Introduction

Colorectal cancer (CRC) is one of the cancer types with the highest morbidity and mortality worldwide, which has a complex tumor microenvironment (TME). The TME contains a variety of immune cells that regulate tumor occurrence and progression, and these immune cells are closely related to the clinical outcomes of cancer patients. For instance, tumor-associated macrophages (TAMs) play a significant role in the remodeling of the TME and cancer progression, highlighting them as promising therapeutic targets [1, 2]. In the TME, TAMs acquire distinct metabolic phenotypes that shape their immunological functions [3–5]. Tumor cells usually produce abnormally high levels of lactic acid, which acts as a metabolic signal that drives macrophages toward an M2-like immunosuppressive state via histone and non-histone protein lactylation [6–9]. However, the identity and biological role of non-histone lactylated enzymes in TAMs remain largely uncharacterized [10, 11].

Long-chain acyl-CoA synthase 3 (ACSL3) is a key enzyme in lipid metabolism that regulates intracellular lipid synthesis, energy metabolism and signal transduction by catalyzing the generation of acyl-CoA esters from long-chain fatty acids [12–14]. It plays an important role in the processes of tumors, metabolic diseases and ferroptosis [15–18]. Recently, we demonstrated that monounsaturated fatty acids promote cancer radioresistance by inhibiting ferroptosis through ACSL3 [19]. Interestingly, ACSL3 was reported to promote macrophage-to-myofibroblast transition in chronic pancreatitis, suggesting its important role in macrophages [20]. However, little is known about the mechanism regulating the activity of ACSL3, especially in immune cells.

Post-translational modifications (PTMs) are one of the most important ways to regulate protein function and are involved in processes such as proliferation, activation, and metabolic reprogramming of immune cells [21–23]. Lactylation is a newly discovered PTM that alters the structure and function of proteins by adding lactyl groups to the lysine residues [6, 11, 24]. Recent studies have revealed that lactylation can affect the function of immune cells, promote tumor immune escape, and thereby influence tumor progression [24–26]. Therefore, we sought to determine whether ACSL3 is regulated by lactylation to support the tumor suppressive phenotype of TAM.

Here, we demonstrated that ACSL3 was highly expressed in TAMs and correlated with poor prognosis in CRC. ACSL3 was delactylated at K679 mediated by SIRT7, which increases its activity, thereby regulating ferroptotic sensitivity and TAM polarization. Inhibition of ACSL3 lactylation in TAMs restored T cell cytotoxicity and suppressed CRC progression. This study may provide new therapeutic targets and strategies for immunotherapy of CRC patients.

## Materials and methods

### Cell culture

All cell lines used in this study, including colorectal cancer cell lines (MC38, HCT116, and SW620), the normal human colonic epithelial cell line (NCM460), the murine macrophage cell line (RAW264.7), the human monocytic cell line (THP-1), and the human T lymphocyte cell line (Jurkat) and the human embryonic kidney cell line (HEK293T) were obtained from the American Type Culture Collection (ATCC, Manassas, VA, USA). All cell lines were authenticated by short tandem repeat (STR) profiling and routinely tested to ensure the absence of mycoplasma contamination before use. MC38, HCT116, NCM460, and RAW264.7 cells were cultured in DMEM (eallbio, 03.1006c, China) containing 10% fetal bovine serum (FBS, Bio Channel, SE-FBS08, China) and 1% penicillin/streptomycin (Beyotime, C0222, China). SW620, THP-1 and Jurkat T cells were cultured in RPMI 1640 medium (eallbio, 03.4007c, China) containing 10% FBS and 1% penicillin/streptomycin. The cells were grown in a humidified cell incubator at 37 °C in a 5% CO_2_ atmosphere. THP-1 monocytes were differentiated into macrophages by incubation with 200 ng/ml phorbol 12-myristate 13-acetate (PMA, Singa, P1585, USA) for 24 h.

### Macrophage–T cell co-culture assay

THP-1 monocytes were differentiated into macrophages by treatment with phorbol 12-myristate 13-acetate (PMA) for 24 h, followed by polarization under the indicated conditions. Jurkat T cells were pre-activated with phytohemagglutinin (Singa, L1668, USA) and concanavalin A (Solarbio, C8110, China) for 24 h, then washed and resuspended in complete RPMI-1640 medium. For co-culture, differentiated macrophages were seeded in the lower chamber of a Transwell system, and activated Jurkat T cells were added to the upper chamber at a ratio of 5:1 (T cells to macrophages). Co-culture was maintained for 48–72 h in complete RPMI-1640 medium supplemented with 10% fetal bovine serum and 1% penicillin-streptomycin at 37 °C with 5% CO₂. Following co-culture, Jurkat T cells were collected from the upper chamber, and the expression of cytotoxic effector genes, including perforin (PRF1) and granzyme B (GZMB), was analyzed by qPCR.

### Clinical samples

Tumor tissues were obtained from patients with colorectal cancer (CRC) undergoing surgical resection for subsequent analysis. As controls, peripheral blood samples were collected from healthy donors. All participants provided written informed consent prior to sample collection. Clinical samples were collected and processed in an anonymized manner. This study was conducted in accordance with the Declaration of Helsinki and the CIOMS guidelines and was approved by the Ethics Committee of the Affiliated Hospital of Jiangnan University (Approval No. LS2025376).

### Isolation of TAMs

Fresh CRC tissues were mechanically minced and enzymatically digested to obtain single-cell suspensions, as previously described [27]. The resulting cells were seeded into culture plates and incubated at 37 °C to allow selective adherence of macrophages. Non-adherent cells were removed by gentle washing with PBS, and the adherent cells, enriched for TAMs, were collected for subsequent analysis. This approach is based on the intrinsic adhesive properties of macrophages.

### Generation of macrophages from PBMCs

Peripheral blood mononuclear cells (PBMCs) were isolated from healthy donors by density gradient centrifugation, as previously described [28]. Monocytes were obtained by adherence to culture plates and subsequently differentiated into macrophages under standard culture conditions.

Protein lysates from TAMs and control macrophages were prepared separately and subjected to western blot analysis.

### Reagents and antibodies

Nicotinamide (NAM, Beyotime, S1761, China), 3×Flag peptides solution (Beyotime, P9801, China), Interleukin-4 (IL-4, STARTER, UA040469, China), Interferon-γ (IFN-γ, STARTER, UA040066, China), Interleukin-13 (IL-13, STARTER, UA040113, China), lipopolysaccharide (LPS, Santa Cruz, sc-3535, USA). Collagenase from Clostridium histolyticum (Singa, C0130, USA), Hyaluronidase from bovine testes (Singa, H3506, USA). The detailed information of all primary antibodies was provided in Supplementary Table 1.

### Plasmid construction

The cDNA encoding full-length human ACSL3 was cloned into Flag-tagged vectors (pRK7-Flag). Point mutations in the ACSL3 constructs were generated using a site-directed mutagenesis kit (C215-01, Vazyme, China).

### Oligonucleotides

The small interfering RNAs (siRNAs) targeting ACSL3 and SIRT7 were purchased from RiboBio (China), and the siRNA sequences are as follows: si SIRT7#1: 5’-CACCTTTCTGTGAGAACGGAA-3’; si SIRT7#2: 5’-TAGCCATTTGTCCTTGAGGAA-3’; si ACSL3#1: 5’-UAACUGAACUAGCUCGAAA-3′; si ACSL3#2: 5’-GCAGUAAUCAUGUACACAA-3’. The sgRNA targeting sequences: Human ACSL3 sgRNA: 5’-AGCTATCATCCACTCGGCCC-3’; Mouse ACSL3 sgRNA: 5’-GAGTCCGGTTTGGAACTGAC-3’.

### Western blotting

Total protein was extracted using RIPA buffer containing protease and phosphatase inhibitors. Equal amounts of protein were separated by SDS-PAGE and transferred to PVDF membranes (IPVH00010, Millipore, USA). Membranes were blocked with 5% non-fat milk for 1 h at room temperature and incubated overnight at 4 °C with primary antibodies. After washing, membranes were incubated with HRP-conjugated secondary antibodies (1:5000) for 1 h. Protein bands were visualized using enhanced chemiluminescence (ECL, E412-02, Vazyme).

### Multiplex immunofluorescence (mIF)

Formalin-fixed paraffin-embedded (FFPE) human CRC tissues and matched normal tissues were sectioned at 4 μm thickness and subjected to mIF using a commercial kit (Formax, mIHC003, China) according to the manufacturer’s instructions. Briefly, sections were incubated with primary antibodies (1:100 dilution), followed by fluorophore-conjugated secondary antibodies, and counterstained with DAPI. Fluorescent images were acquired using a confocal laser scanning microscope under identical acquisition settings. For murine samples, colorectal tissues from the sgCtrl and sgACSL3 groups were processed in parallel using the same staining protocol. IF staining of cultured cell coverslips was performed using an identical procedure. All staining results were independently evaluated by two investigators blinded to sample identity.

### Immunohistochemistry (IHC)

Ki67 staining was performed on formalin-fixed paraffin-embedded colorectal cancer tissues according to standard immunohistochemistry protocols. Briefly, tissue sections were processed using a commercial immunohistochemistry kit (Gene Technology, GK700610, China) following the manufacturer’s instructions. Sections were incubated with anti-Ki67 antibody (1:100 dilution), followed by HRP-conjugated secondary antibody and visualized using DAB as the chromogen.

### Cell viability assay

Cell viability was assessed using a Cell Counting Kit-8 (CCK-8) assay (ApexBio, K1018, USA). HCT116 and SW620 cells were trypsinized, counted, and seeded into 96-well plates at a density of approximately 2000-3000 cells per well, with three replicate wells per condition. After cell attachment, the culture medium was replaced with conditioned medium derived from the TAM co-culture system for treatment. At designated time points, 10 μL of CCK-8 reagent was added to each well, followed by incubation at 37 °C in the dark for approximately 1.5 h. Absorbance was measured at 450 nm using a microplate reader. Cell proliferation was monitored for 5 consecutive days, and growth curves were generated based on OD450 values.

### Colony formation ability

HCT116 cells were seeded into 6-well plates at a density of approximately 1000 cells per well. After adherence, cells were treated with TAM-conditioned medium and cultured for 14 days to allow colony formation. Colonies were fixed with 4% paraformaldehyde for 30 min at room temperature, washed with phosphate-buffered saline (PBS), and stained with crystal violet (Beyotime, C0121, China) for 15 min. Excess dye was removed with PBS, and plates were air-dried prior to imaging. Colonies were photographed and quantified.

### Co-immunoprecipitation (Co-IP)

Cells were washed with phosphate-buffered saline (PBS) to remove culture medium and lysed in ice-cold NP-40 lysis buffer (50 mM Tris-HCl, pH 7.5, 150 mM NaCl, 0.1–0.5% NP-40, 1 mM PMSF, and protease inhibitors). Cell lysates were incubated on a 4 °C shaker for 30 min and subsequently centrifuged at 13,000 rpm for 5 min at 4 °C to remove insoluble debris. A portion of the supernatant (50 μL) was collected as input and mixed with 5× SDS loading buffer, followed by heating at 95 °C for 10 min and brief centrifugation. Input samples were stored at −80 °C for further analysis. The remaining supernatant was incubated with anti-Flag M2 magnetic beads (Sigma, USA; M2283) at 4 °C with gentle rotation for 3 h. After incubation, beads were washed three times with NP-40 lysis buffer to eliminate nonspecific binding. Immunocomplexes were eluted by boiling in 1× SDS loading buffer for 10 min and subsequently analyzed by western blotting.

### Quantitative real-time PCR (qPCR)

Total RNA was extracted using TRIzol reagent (Yeasen, 10606ES60, China) and reverse transcribed into cDNA using a PrimeScript RT Reagent Kit (Vazyme, E303-01, China). qPCR was performed using SYBR Green Master Mix (Yeasen, 11201ES08, China) on a QuantStudio 6 Flex Real-Time PCR System. β-actin served as an internal control. Relative gene expression was calculated using the 2^–ΔΔCt^ method. Gene-specific primers were synthesized by Genewiz (China). The detailed information of qPCR primers was provided in Supplementary Table 2.

### Flow cytometry

Macrophages were stained with fluorophore-conjugated monoclonal antibodies against HLA-DR (eBioscience, 11-9956-42, USA) and CD206 (eBioscience, 17-2069-42, USA) according to the manufacturer’s instructions. Following staining, cells were washed and subjected to flow cytometric analysis using a BD FACSCanto II or Agilent ACEA NovoCyte flow cytometer. Data were analyzed using FlowJo software (Tree Star, USA).

### Lipid ROS assay

Lipid reactive oxygen species (ROS) levels were measured using the fluorescent probe BODIPY 581/591 C11 (MCE, HY-D1301, USA). Following the indicated treatments, cells were incubated with 5 μM BODIPY 581/591 C11 at 37 °C for 30 min in the dark according to the manufacturer’s protocol. Fluorescence intensity was then quantified by flow cytometry, and data were analyzed with FlowJo V10 software.

### Measurement of malondialdehyde (MDA)

MDA levels were determined using a commercial assay kit (Beyotime, S0131S, China) according to the manufacturer’s protocol. Briefly, lysates from cells were centrifuged to obtain the supernatant. The MDA working solution was then added, and samples were incubated at 100 °C for 15 min. After cooling and centrifugation, the absorbance of the supernatant was measured at 532 nm using a microplate reader.

### Measurement of the GSH/GSSG ratio

Intracellular glutathione levels were measured using a commercial assay kit (Beyotime, S0053, China) according to the manufacturer’s instructions. TAMs were collected and lysed, and reduced glutathione (GSH) and oxidized glutathione (GSSG) levels were determined separately following the kit protocol. The GSH/GSSG ratio was calculated based on the measured values of GSH and GSSG to evaluate the cellular redox status.

### Measurement of ferrous iron (Fe²^+^)

Intracellular Fe²^+^ levels were measured using a commercial assay kit (Beyotime, S1066S, China) according to the manufacturer’s instructions. TAMs were collected and lysed, and Fe²⁺ levels were determined based on the colorimetric reaction described in the kit protocol. Absorbance was measured using a microplate reader, and Fe²^+^ levels were calculated according to the standard curve provided by the manufacturer.

### Measurement of ACSL3 activity

ACSL3 enzymatic activity was measured as previously described [29, 30]. Equal amounts of protein were mixed with Medium I (250 mM sucrose (Merck, V900116, USA), 10 mM Tris (Sangon Biotech, A501492, China) (pH 7.4), 1 mM EDTA (MCE, HY-Y0682, USA), 1 mM DTT (MCE, HY-15917A, USA)). The reaction mixture (120 μL) containing 125 mM Tris-HCl (pH 7.5), 10 mM ATP (MCE, HY-B2176, USA), 10 mM MgCl₂ (Sangon Biotech, A631017, China), 0.25 mM CoA (MCE, HY-128851, USA), 5 mM DTT, 0.5 mM Triton X-100, and 0.05 mM BODIPY 500/510 C4,C9 (Thermo Fisher Scientific, D3821, USA) was added to 80 μL of Medium I and incubated at 37 °C for 30 min. Reactions were terminated with 1 mL of Dole’s reagent (isopropanol: heptane: H₂SO₄ = 40: 10: 1), followed by two heptane washes (2 mL each). The aqueous phase containing BODIPY-CoA was collected, and fluorescence intensity was determined using a Synergy HT microplate reader (BioTek, USA).

### Generation of reconstituted THP-1 cell lines

To generate stable reconstituted lines, sgRNA targeting the endogenous ACSL3 was cloned into pLKO.1 vector containing a puromycin selection marker. sgRNA-resistant wild-type (WT) or K679R/K679Q ACSL3 cDNA was inserted into pLV vectors containing a neomycin selection marker. Recombinant lentiviruses were produced by Guangzhou Packgene Co., Ltd. (China) and used to infect THP-1 cells. Stable clones were selected using puromycin or neomycin, differentiated into TAMs, and verified for protein expression by western blotting.

### Mouse tumor models

MC38 tumor cells were mixed with RAW264.7 macrophages (sgCtrl or sgACSL3) at a 1:1 ratio prior to implantation.

Orthotopic colorectal tumor model: For orthotopic implantation, 6-week-old male C57BL/6 mice were anesthetized, and a small midline abdominal incision was made under sterile conditions to expose the cecum and colon. The mixed cell suspension was injected into the subserosal layer of the colon wall using a microsyringe. Successful injection was confirmed by the formation of a localized bleb. The needle was carefully withdrawn to prevent leakage, and the intestine was returned to the abdominal cavity. The peritoneum and skin were sutured in layers. Mice were monitored during recovery and maintained under standard housing conditions. Fifteen days after implantation, mice were euthanized, and tumor tissues were collected for further analysis.

Subcutaneous tumor model: For subcutaneous implantation, the mixed cell suspension was injected into the axillary region of 6-week-old male C57BL/6 mice. Tumor dimensions were measured every 3 days using digital calipers, and tumor volume was calculated as (length × width²)/2. Eighteen days after implantation, mice were euthanized, tumors were excised and weighed, and samples were collected for downstream analyses.

All procedures were approved by the Ethics Committee of the Affiliated Hospital of Jiangnan University and performed in accordance with institutional guidelines for the care and use of laboratory animals (JN.No20250415c0400731 [211]).

### Statistical analyses

All data are presented as the mean ± standard deviation (SD). Statistical significance between two groups was determined using a two-tailed Student’s *t* test, while comparisons among multiple groups were analyzed by one-way ANOVA. Survival curves were generated using the Kaplan–Meier method and compared with the log-rank test. Univariate and multivariate Cox proportional hazards models were applied to identify independent prognostic factors. Statistical analyses were conducted using SPSS software version 20.0 (IBM, USA), and *P* < 0.05 was considered statistically significant. Publicly available datasets from the GEO database were analyzed using R (version 4.3). Data processing and visualization were performed using standard R packages.

## Results

### ACSL3 is highly expressed in TAMs and correlates with poor prognosis in CRC

**Fig. 1:**
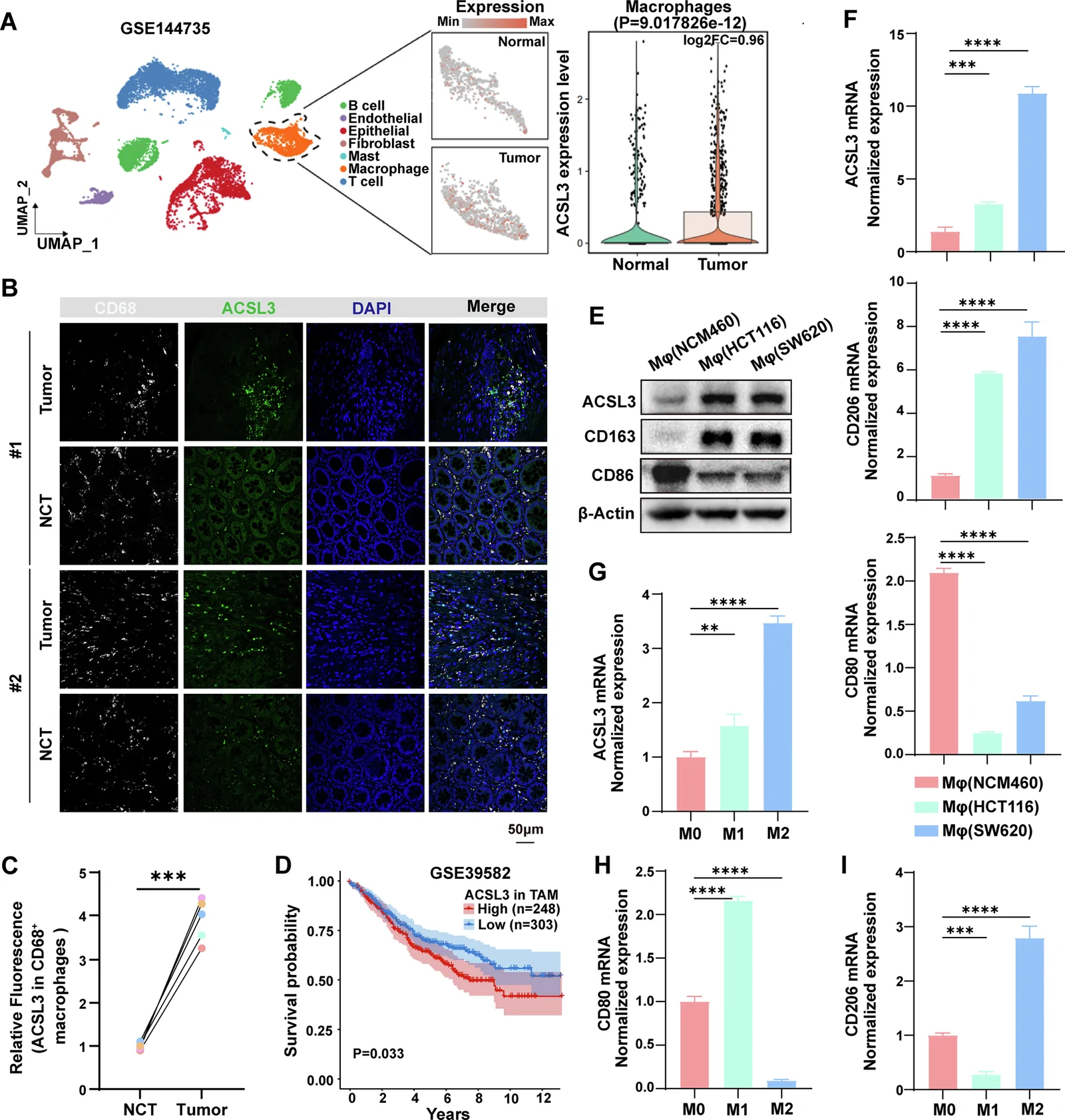
ACSL3 is highly expressed in TAMs and correlates with poor prognosis in CRC. **A** Single-cell RNA-seq data (GSE144735) from colorectal cancer tissues were analysed by UMAP to identify major cell populations. ACSL3 expression across cell subsets was visualized, with comparison between normal and tumor samples (two-sided Wilcoxon rank-sum test). **B** Representative IF images of human CRC tissue adjacent non-tumorous tissues showed co-localization of ACSL3 (green) with CD68^+^ (white); nuclei were stained with DAPI (blue). Scale bar, 50 μm. **C** Quantitative statistical analysis was performed on the multiplex immunofluorescence results. *n* = 5. **D** Kaplan–Meier overall-survival analysis of CRC patients in the GSE39582 cohort stratified by ACSL3 expression in TAMs. P values were calculated using the log-rank test (Mantel-Cox), *P* = 0.033. **E**, **F** Western blotting and qRT–PCR analyses were performed to examine ACSL3 and macrophage markers in NCM460 epithelial cells and HCT116/SW620 CRC lines co-cultured with monocyte-derived macrophages (MΦ). **G**–**I** qRT–PCR analyses of relative ACSL3 mRNA levels and subtype-specific marker expression in distinct macrophage populations. Data are presented as mean ± SD. Statistical significance was determined using a two-tailed paired Student’s *t* test (C) and one-way ANOVA followed by Tukey’s multiple comparisons test (**G**–**I**). **P < 0.01, ***P < 0.001, ****P < 0.0001.

First, we analyzed the characteristics of the TME based on a single-cell transcriptomic analysis dataset (GSE144735 and GSE132257), and revealed significant clustering of immune and matrix populations in CRC tissues. Further analysis showed that TAMs displayed prominently elevated ACSL3 expression relative to macrophages in normal mucosa samples (Fig. 1A and Supplementary Fig. S1A). To confirm the upregulation of ACSL3 in CRC TAMs, we detected ACSL3 and CD68 levels in CRC tissues by mIF, and found that ACSL3 was enriched in TAMs compared with matched NCTs (*P* < 0.001, Fig. 1B, C). We used the ssGSEA algorithm to calculate the relative ACSL3 expression of TAMs in the GEO database (GSE39582) of the CRC cohort, which showed that patients with higher ACSL3 in TAMs exhibited poorer overall survival (Fig. 1D). Together, these results indicate that ACSL3 is increased in TAMs and correlates with poorer prognosis in CRC patients.

Then, macrophages were co-cultured with CRC cells or normal epithelial NCM460 cells, and significant upregulation of ACSL3 was observed in the CRC cells co-cultured group (Fig. 1E, F). qRT–PCR profiling of macrophage subsets demonstrated that elevated ACSL3 expression was accompanied by increased M2-associated markers (Fig. 1F–I and Supplementary Fig. S1B–E). Consistently, flow cytometry analysis confirmed the successful induction of macrophage polarization phenotypes (Supplementary Fig. S1F, G). Collectively, these data demonstrate that ACSL3 is preferentially upregulated in TAMs and correlates with an immunosuppressive phenotype and unfavorable prognosis in CRC.

### Inhibition of ACSL3 in TAMs enhances antitumor activity

**Fig. 2:**
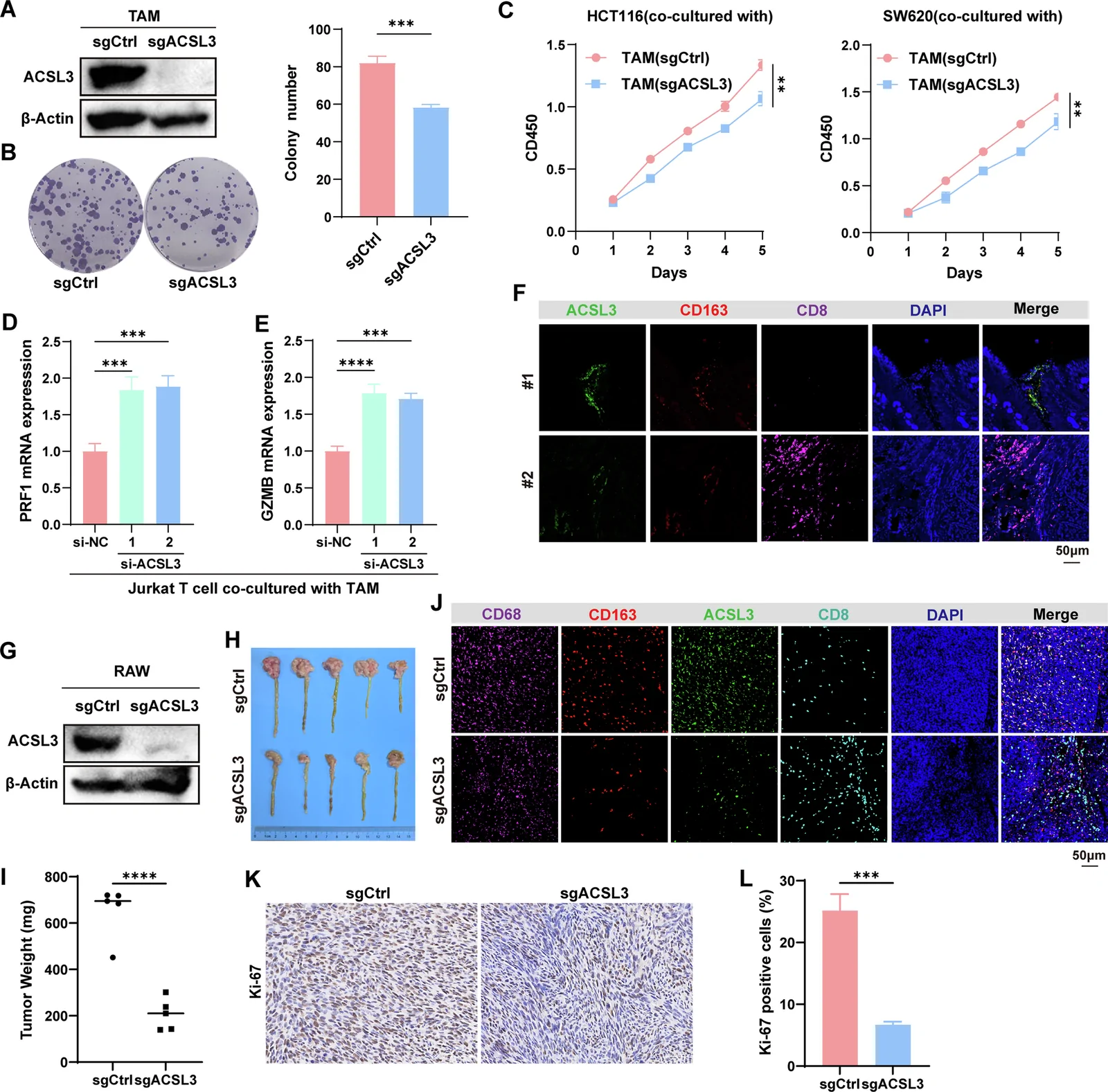
Inhibition of ACSL3 in TAMs enhances antitumor activity. **A** Western blotting analysis was performed to verify ACSL3 knockout in THP-1–derived macrophages (sgACSL3). **B** Colony formation assays were conducted to assess the proliferative capacity of HCT116 cells co-cultured with TAMs after ACSL3 inhibition. **C** CCK-8 assay assessing proliferation of various CRC cell lines under similar conditions. **D**, **E** ACSL3-deficient TAMs were co-cultured with T cells, and the expression of effector factors (GZMB, PRF1) in t cells was detected by qRT-PCR analysis. **F** MIF staining of human CRC tissue sections to visualize ACSL3 expression, CD163^+^ TAMs, and CD8 ^+^ T-cell distribution. Scale bar, 50 μm. **G** Western blotting validation of ACSL3 knockout in RAW264.7 cells (sgACSL3). **H** Representative tumor images from an orthotopic CRC model established by co-injection of MC38 cells and RAW264.7-derived macrophages (1:1). **I** Endpoint tumor weight measurements in mice with or without ACSL3-deficient macrophages. **J** mIF staining of tumor tissues showing ACSL3 expression, CD68^+^ and CD163^+^ macrophage infiltration, and CD8^+^ T-cell distribution. Scale bar, 50 μm. **K**, **L** IHC analysis and quantification of Ki-67 expression in tumor tissues from C57BL/6 mice with or without ACSL3 knockout. Scale bar, 50 μm. Data are presented as mean ± SD from three independent experiments unless otherwise indicated. Statistical significance was determined using a two-tailed Student’s *t* test for comparisons between two groups (**B**, **I**, **L**), one-way ANOVA followed by Tukey’s multiple comparisons test for multiple group comparisons (**D**, **E**), and two-way ANOVA for time-course analyses (**C**). **P < 0.01, ***P < 0.001, ****P < 0.0001.

In order to investigate the role of ACSL3 in TAMs, stable ACSL3 knockout was achieved in THP-1 cells via CRISPR/Cas9-mediated gene editing (sgACSL3), as confirmed by western blotting (Fig. 2A). Co-culture with ACSL3-deficient TAMs significantly suppressed the proliferation of HCT116 CRC cells in colony formation and CCK-8 assays compared with control TAMs (Fig. 2B, C). TAMs with ACSL3 knockdown significantly elevated the expression of GZMB and PRF1 in T cells (Fig. 2D, E), suggesting that ACSL3 depletion potentiated T cell cytotoxicity. In human CRC tissues, mIF staining demonstrated strong co-localization of ACSL3 with CD163⁺ TAMs and an inverse association with CD8^+^ T cell infiltration (Fig. 2F).

Parallel findings were validated in RAW264.7 macrophages with stable ACSL3 knockout (Fig. 2G). We next evaluated the in vivo relevance of these observations using two complementary tumor models. In an orthotopic model, co-injection of RAW264.7 macrophages and MC38 cells (1:1 ratio) into the colon revealed that ACSL3-deficient macrophages markedly suppressed tumor growth compared with control groups (Fig. 2H). Tumor weight was significantly reduced (Fig. 2I), and immunohistochemical analysis showed decreased Ki-67 staining, indicating reduced proliferative capacity (Fig. 2K, L). To further substantiate these results, a subcutaneous tumor model was employed. Consistently, co-injection of ACSL3-deficient macrophages led to a significant reduction in tumor volume and weight compared with controls (Supplementary Fig. S2B–D), while no significant difference in body weight was observed (Supplementary Fig. S2E), suggesting minimal systemic toxicity. In line with these findings, mIF analysis of tumor sections revealed that tumors derived from ACSL3-deficient macrophages exhibited decreased infiltration of CD163^+^ M2-like macrophages and enhanced accumulation of CD8^+^ T cells (Fig. 2J and Supplementary Fig. S2H), further validating that ACSL3 fosters an immunosuppressive TME.

Together, these results demonstrated that ACSL3 in TAMs promotes CRC progression by fostering an immunosuppressive and tumor-supportive microenvironment.

### SIRT7 functions as the principal de-lactylase of ACSL3

**Fig. 3:**
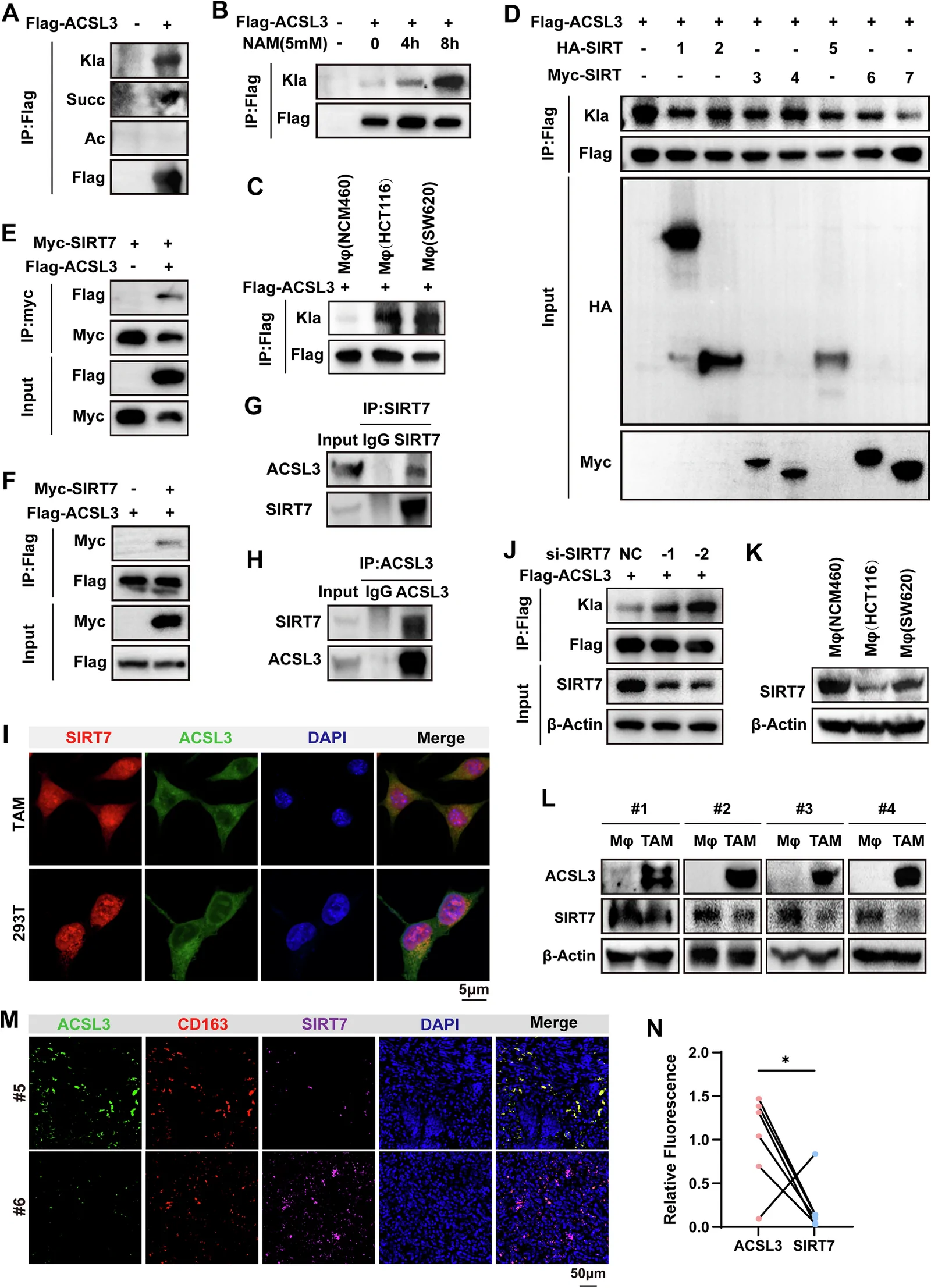
SIRT7 functions as the principal de-lactylase of ACSL3. **A** Co-IP and western blotting were performed to examine acylation modifications of ACSL3. **B** Time-dependent accumulation of ACSL3 lactylation in response to pan-Sirtuin inhibitor nicotinamide (NAM). **C** ACSL3 lactylation was evaluated in macrophages co-cultured with CRC cells (HCT116, SW620) or normal epithelial cells (NCM460). **D** Overexpression of individual Sirtuin family members was used to assess their effects on ACSL3 lactylation. **E**–**H** Reciprocal co-IP assays were carried out to detect the interaction between ACSL3 and SIRT7. **I** IF staining was performed to visualize ACSL3 (green) and SIRT7 (red) localization in TAMs and HEK293T cells; nuclei were stained with DAPI (blue). Scale bar, 5 μm. **J** SIRT7 knockdown cells were subjected to Flag-ACSL3 immunoprecipitation followed by Kla immunoblotting to assess ACSL3 lactylation. **K** SIRT7 expression was examined in macrophages co-cultured with colorectal cancer cells or epithelial control cells. **L** Western blotting of ACSL3 in patient-derived TAMs and control macrophages from four CRC tissue donors (#1–4). **M**, **N** mIF staining of human CRC tissues showing ACSL3, CD163, and SIRT7 expression, and quantification of ACSL3 and SIRT7 fluorescence intensity in TAMs. Scale bar, 50 μm. Data are presented as mean ± SD (*n* = 6). *P < 0.05 (two-tailed Student’s *t* test).

Besides transcriptional regulation, PTMs are the most crucial methods for regulating metabolic enzymes. They can promptly and rapidly modify specific sites on metabolic enzymes, enabling cells to swiftly respond to changes in both internal and external environments. To elucidate the regulatory mechanism behind ACSL3, we utilized co-IP and western blotting analysis, which revealed multiple acyl modifications of ACSL3, with lactylation being the predominant one (Fig. 3A). Treatment with the pan-sirtuin inhibitor nicotinamide (NAM) induced a time-dependent accumulation of lactylated ACSL3, suggesting Sirtuin-dependent de-lactylation (Fig. 3B).

The lactylation level of ACSL3 in TAMs was observed to be significantly higher than that in macrophages co-cultured with normal epithelial cells (Fig. 3C). To explore the deacetylase isoforms that modify ACSL3, seven sirtuin isoforms were tested and only SIRT7 effectively reduced ACSL3 lactylation, indicating its substrate specificity (Fig. 3D). Reciprocal co-IP assay confirmed a physical interaction between ACSL3 and SIRT7 (Fig. 3E–H), and IF imaging demonstrated their cytoplasmic co-localization in both TAMs and HEK293T cells (Fig. 3I). The knockdown of SIRT7 significantly increased the lactylation of ACSL3 (Fig. 3J). In addition, compared with the control group, the expression of SIRT7 in TAMs co-cultured with CRC cells was decreased (Fig. 3K). To further elucidate the clinical relevance of SIRT7 and ACSL3, we examined their expression in CRC TAMs by western blotting. The results revealed that ACSL3 expression was significantly elevated in TAMs compared with macrophages derived from peripheral blood monocytes of healthy donors, whereas SIRT7 expression was markedly decreased (Fig. 3L). MIF analysis further visualized the distribution of ACSL3 and SIRT7 in CD163^+^ TAMs (Fig. 3M), and quantitative analysis validated their inverse correlation (Fig. 3N). These data collectively identify SIRT7 as the primary de-lactylase responsible for ACSL3 regulation in TAMs.

### Lactylation at K679 enhances ACSL3 enzymatic activity

**Fig. 4:**
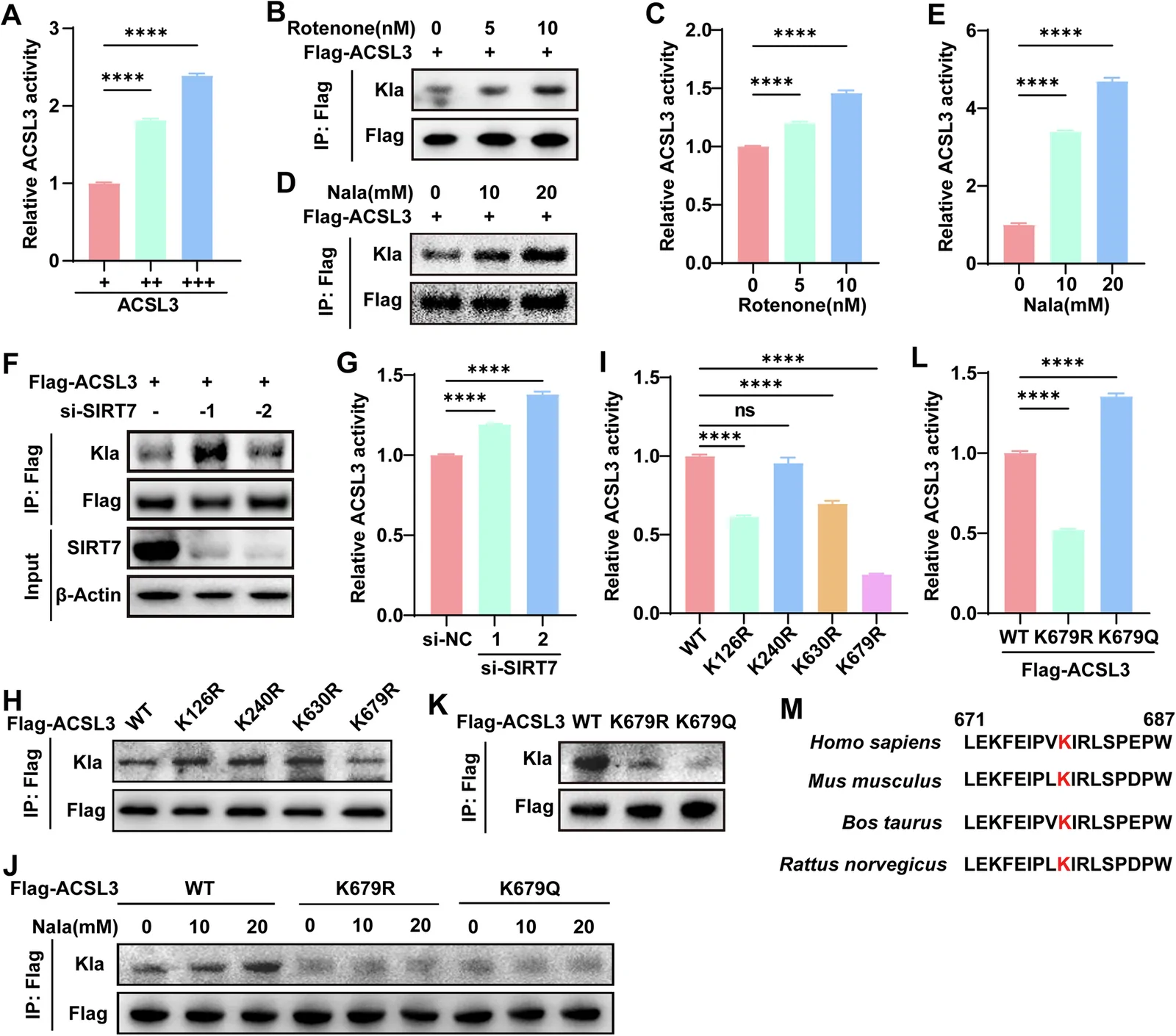
ACSL3 K679 lactylation enhances its enzymatic activity. **A** In vitro enzymatic assay in HEK293T cells showed ACSL3 catalytic activity increases in a dose-dependent manner, illustrating assay robustness. **B**, **C** Western blotting and enzymatic assays were conducted to examine ACSL3 lactylation and catalytic activity following rotenone treatment at indicated concentrations (0, 5, 10 nM). **D**, **E** Western blotting and enzymatic analyses were performed to measure ACSL3 lactylation and enzymatic activity in HEK293T cells treated with exogenous lactate (0, 10, 20 mM). **F**, **G** Western blotting and enzymatic assays were used to evaluate ACSL3 lactylation and enzymatic activity in Flag-ACSL3-expressing cells after SIRT7 knockdown using two independent siRNAs (#1, #2). **H**, **I** Western blotting and enzymatic assays were performed to compare WT ACSL3 with the K126R, K240R, K630R, and K679R mutants in HEK293T cells. **J** Immunoblot analysis of lactylation levels of ACSL3 WT, K679R, and K679Q under increasing concentrations of sodium lactate. **K**, **L** Western blotting and enzymatic assays were performed to compare WT ACSL3 with the K679R and K679Q mutants in HEK293T cells. **M** Sequence alignment was conducted to examine the conservation of ACSL3 K679 across multiple species. Data are presented as mean ± SD (*n* = 3). Statistical significance was determined by one-way ANOVA with Tukey’s multiple comparisons test (**A**, **C**, **E**, **G**, **I**, **L**). Comparisons were performed relative to control (**C**, **E**) or WT (**I**, **L**). ****P < 0.0001, ns, not significant.

After confirming that SIRT7 mediates delactylation of cytoplasmic ACSL3, we proceeded to examine how this modification influences ACSL3 enzymatic function. To assess its activity, a fluorescence-based assay was employed, in which lactate was conjugated with the fluorescent probe BODIPY (4,4-difluoro-3a, 4a-diaza-s-indacene) to generate a labeled substrate, as described in previous studies [29, 30]. To quantify ACSL3 catalytic function, an in vitro enzymatic assay was established in HEK293T cells, and the results showed that its activity was dose-dependent (Fig. 4A). Mitochondrial inhibition by rotenone, which elevates intracellular lactate, enhanced ACSL3 lactylation and enzymatic activity in a dose-dependent manner (Fig. 4B, C). Similarly, exogenous lactate supplementation (0–20 mM) promoted both ACSL3 lactylation and activity (Fig. 4D, E).

Moreover, silencing SIRT7 augmented ACSL3 lactylation and concomitantly increased its enzymatic activity (Fig. 4F, G), indicating that SIRT7-mediated de-lactylation is essential for catalytic efficiency. Site-directed mutagenesis identified K679 as the major lactylation site, because the K679R mutant exhibited drastically reduced lactylation compared with ACSL3 WT (Fig. 4H, I). To assess the lactate responsiveness of ACSL3 lactylation, ACSL3 WT, K679R, and K679Q were evaluated across increasing concentrations of sodium lactate. ACSL3 WT displayed a marked dose-dependent increase in lactylation, whereas both K679R and K679Q mutants maintained low basal levels with minimal to no induction (Fig. 4J). Further mutational analysis revealed that the de-lactylation mimic K679R exhibited diminished activity, whereas the lactylation mimic K679Q significantly increased enzymatic function relative to the ACSL3 WT (Fig. 4K, L). Sequence alignment confirmed that K679 is highly conserved among species (Fig. 4M).

These results collectively establish K679 lactylation as a critical determinant of ACSL3 enzymatic activation.

### ACSL3 inhibition in TAMs induces ferroptosis and reprograms macrophages toward an M1-like phenotype

**Fig. 5:**
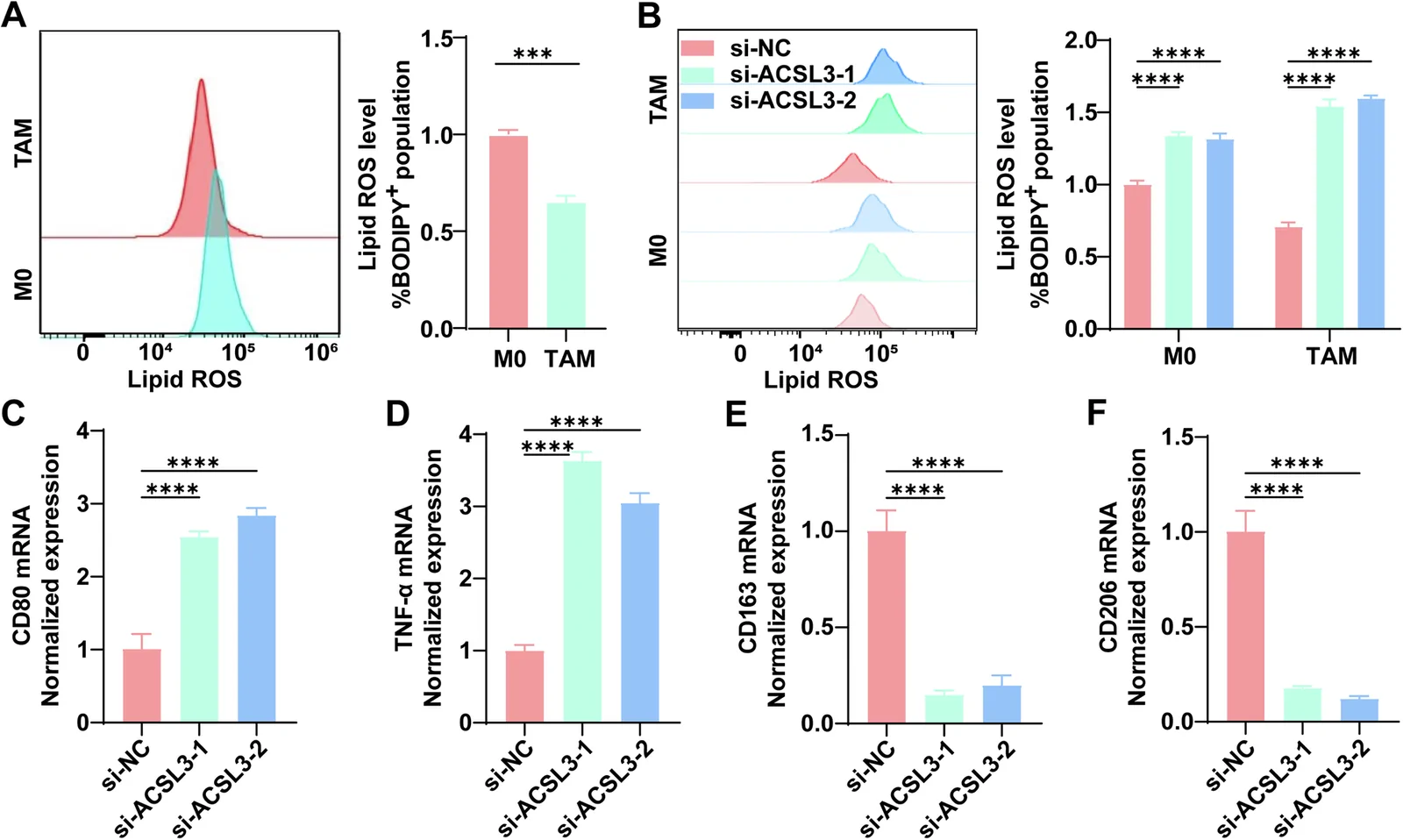
ACSL3 inhibition in TAMs induces ferroptosis and reprograms macrophages toward an M1-like phenotype. **A** Flow cytometric analysis of lipid ROS levels in M0 macrophages and TAMs; quantitative results are shown on the right. **B** Flow cytometric analysis and quantification of lipid ROS levels in TAMs (HCT116 + MΦ) and M0 macrophages transfected with ACSL3-specific siRNAs (siACSL3-1, siACSL3-2) or negative control siRNA (siNC). **C**–**F** THP-1 monocytes differentiated into M0 macrophages using PMA and subsequently polarized into TAMs by co-culture with HCT116 cells, then transfected with ACSL3-specific or control siRNA. qRT–PCR quantifies expression of M1 markers (CD80, TNF-α) and M2 markers (CD163, CD206). Data are presented as mean ± SD (*n* = 3). Statistical significance was determined using a two-tailed Student’s t-test for comparisons between two groups (**A**), one-way ANOVA followed by Tukey’s multiple comparisons test for multiple group comparisons (**C**–**F**), and two-way ANOVA for (**B**). ***P < 0.001, ****P < 0.0001.

Recently, we have demonstrated that ACSL3 plays an important role in mediating ferroptosis resistance in radioresistant cancer cells [19]. Several studies have revealed the significance of ferroptosis in the phenotype of macrophages. The immunosuppressive M2 phenotype is sensitive to ferroptosis, while M1-type macrophages are resistant to ferroptosis [31–33]. Given the preferential expression of ACSL3 in TAMs, we explored the effect of ACSL3 on ferroptosis and the phenotype of TAM. The functional correlation was studied through targeted silencing technology. Flow cytometry results showed that knockdown of ACSL3 significantly increased the accumulation of lipid reactive oxygen species (ROS) in TAMs, indicating enhanced lipid peroxidation and ferroptosis stress (Fig. 5A, B).

Meanwhile, ACSL3 depletion reprogrammed TAMs from an M2-like to an M1-like phenotype, as evidenced by upregulation of M1-associated markers (CD80, TNF-α, iNOS, IL-1β, and IL-6) and concomitant downregulation of M2-associated markers (CD163, CD206, Arg1, IL-10, and TGF-β) (Fig. 5C–F and Supplementary Fig. S2A–F). This phenotypic transition was accompanied by elevated transcription of M1-associated cytokines such as TNF-α and a reduction of M2 markers, including CD206, further confirming the pro-inflammatory polarization shift. These findings identify ACSL3 as a key metabolic regulator that suppresses ferroptosis and maintains the M2 immunosuppressive phenotype of TAMs.

### ACSL3 K679 lactylation confers ferroptosis resistance in TAMs and promotes tumor progression

**Fig. 6:**
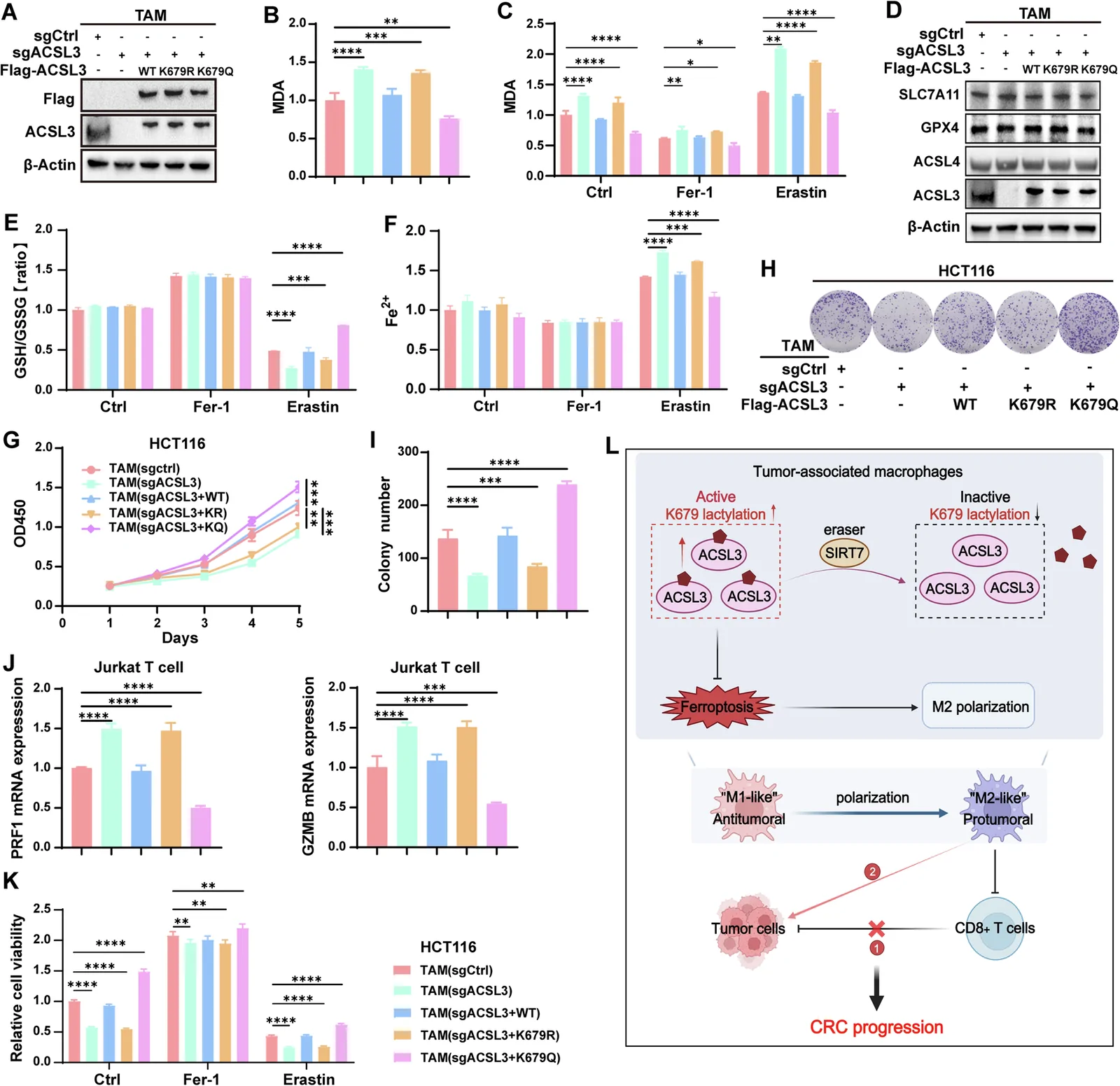
ACSL3 K679 lactylation confers ferroptosis resistance in TAMs and drives tumor progression. **A** Western blotting validation of Flag-tagged ACSL3 expression in THP-1–derived macrophages with ACSL3 knockout (sgACSL3) and reconstitution with WT, K679R or K679Q mutants. **B** Quantification of MDA levels in TAMs. TAMs with sgCtrl, sgACSL3, WT, K679R, or K679Q constructs were analyzed for lipid peroxidation using an MDA assay. Data represent mean ± SD from three independent experiments. **C** MDA levels in the five groups following treatment with ferroptosis inhibitor ferrostatin-1 (Fer-1, 2 µM, 8 h) or ferroptosis inducer erastin (10 µM, 24 h). **D** Immunoblot analysis of GPX4, SLC7A11, ACSL4, and ACSL3 protein expression in TAMs co-cultured with HCT116 cells following ACSL3 depletion. **E** Measurement of GSH/GSSG ratios in TAMs co-cultured with HCT116 cells under the indicated treatments (control, Fer-1, or erastin). **F** Quantification of intracellular Fe²^+^ levels in TAMs co-cultured with HCT116 cells under the indicated treatments (control, Fer-1, or erastin). **G**–**I** Effects of co-culture with TAMs on HCT116 cell proliferation determined by CCK-8 and colony formation assays. **J** qRT–PCR analysis of cytotoxic T-cell effector markers (GZMB, PRF1) following co-culture of T cells with TAMs expressing different ACSL3 variants. **K** Cell viability assays of HCT116 cells co-cultured with TAMs and treated with Fer-1 or erastin. **L** Schematic illustration showing that lactylation at ACSL3 K679 maintains its enzymatic activation in TAMs, leading to ferroptosis resistance and sustaining an M2-like immunosuppressive phenotype. SIRT7-mediated delactylation inactivates ACSL3, thereby enhancing ferroptotic sensitivity and promoting M1-like pro-inflammatory polarization. Functionally, ACSL3 K679 lactylation drives TAM-induced CD8^+^ T-cell suppression and supports colorectal cancer progression, whereas its inhibition restores antitumor immunity. Data are presented as mean ± SD (*n* = 3). Statistical analysis was performed using two-way ANOVA with Tukey’s multiple comparisons test. *P < 0.05, **P < 0.01, ***P < 0.001, ****P < 0.0001.

To determine the functional relevance of ACSL3 K679 lactylation in macrophage-mediated tumor progression, endogenous ACSL3 was depleted and reconstituted with sgRNA-resistant Flag-tagged WT, K679R, or K679Q mutants in THP-1 cells (Fig. 6A). MDA revealed that ACSL3 loss markedly increased lipid peroxidation, whereas WT re-expression restored baseline levels. Among all groups (sgCtrl, sgACSL3, WT, K679R, and K679Q), the de-lactylation mimic K679R showed the highest MDA accumulation, while the lactylation mimic K679Q exhibited minimal lipid peroxidation (Fig. 6B). These data indicate that K679 lactylation protects TAMs from ferroptotic stress.

Further treatment with the ferroptosis inhibitor Fer-1 (Ferrostain-1) reduced the accumulation of MDA, while the ferroptosis inducer erastin exacerbated the accumulation of MDA (Fig. 6C). To determine whether ACSL3 exerts its function through the canonical GPX4–SLC7A11–ACSL4 axis, we examined the expression of key ferroptosis regulators. Neither ACSL3 depletion nor K679 mutants altered GPX4, SLC7A11, or ACSL4 protein levels (Fig. 6D), indicating that ACSL3 acts independently of both the GPX4–GSH pathway and ACSL4-mediated lipid remodeling. Consistently, no differences in GSH/GSSG ratios were observed under basal conditions, Fer-1 treatment increased the ratio across all groups without significantly affecting intergroup differences, whereas erastin treatment decreased the ratio, with these changes paralleling the levels of lipid peroxidation (Fig. 6E). In line with these observations, Fe^2+^ levels remained comparable under control and Fer-1 treatment but increased in response to erastin—an effect consistent with ferroptotic activity (Fig. 6F). The CCK-8 and colony formation experiments demonstrated that co-culture with WT or K679Q TAMs promoted the proliferation of HCT116 cells, while co-cultured with sgACSL3 or K679R TAMs inhibited CRC cell proliferation (Fig. 6G–I). Co-culture experiments with T cells revealed that ACSL3 depletion in TAMs elevated GZMB and PRF1, whereas K679 lactylation reversed this effect (Fig. 6J). Moreover, cell viability assays showed that Fer-1 rescued, whereas erastin further decreased tumor cell survival when co-cultured with ACSL3-deficient TAMs (Fig. 6K).

Together, these findings indicate that ACSL3 K679 lactylation endows TAMs with ferroptosis resistance, maintains their immunosuppressive phenotype, and promotes CRC progression.

## Discussion

The metabolic regulation of immune cells is a crucial link in the immune response, which affects the activation, differentiation and function of immune cells. TAMs are an important component of the TME and can present pro-inflammatory M1-like phenotypes or immunosuppressive M2-like phenotypes based on environmental cues [34]. Metabolic reprogramming is increasingly regarded as a key determinant of TAM polarization [5, 35, 36]. Tumor-derived metabolites (such as lactic acid) accumulate in the microenvironment and drive epigenetic and post-translational modifications of immune cells [37, 38]. Although histone lactylation has been deeply studied, non-histone lactylation is poorly understood, especially the non-histone lactylation modification of immune cells. In the present study, we found that ACSL3 is highly expressed in TAMs of colorectal cancer and promotes tumor growth. Importantly, we found that ACSL3 underwent lactylation at the K679 site and its activity increased, while SIRT7-mediated delactylation of ACSL3 K679 could reduce ACSL3 activity, promote ferroptosis of TAMs and repolarization towards M1, thereby restoring their inhibition of T cells (Fig. 6L). These findings reveal a previously unrecognized role of non-histone lactylation in regulating TAM function.

PTMs are one of the most important and direct means of regulating protein functions. Lactylation modification is a novel post-translational modification that alters the structure and function of proteins by adding lactyl groups to the lysine residues of proteins [6, 11]. lactylation plays a significant role in the occurrence and development of tumors. By influencing the post-translational states of histones and non-histones, it participates in gene expression regulation and cellular signal transduction, thereby affecting the metabolic pathways and immune evasion mechanisms of tumor cells [38]. Lactylation modification has also been found to promote tumor immune escape by influencing the function of immune cells, thereby affecting tumor progression [39–41]. For example, tumor metabolite lactate can promote tumorigenesis by modulating regulatory T cells [24]. Lactylation-driven METTL3-mediated RNA m6A modification was verified to promote immunosuppression of tumor-infiltrating myeloid cells [42]. Here, we clarified the impact of lactylation modification in TAMs on the immunosuppressive TME and investigated the abnormal characteristics of immune cells in the TME from the perspective of lactylation modification, providing a new direction for tumor treatment.

ACSL3 is a central enzyme in lipid metabolism that catalyzes the activation of long-chain fatty acids into acyl-CoA, enabling their incorporation into lipid droplets and other metabolic pathways. Beyond its canonical metabolic roles, ACSL3 modulates ferroptosis sensitivity by controlling cellular lipid composition, thus influencing susceptibility to lipid peroxidation [12–17]. However, the function of ACSL3 in immune cells has not yet been clarified. In this study, we discovered for the first time that ACSL3 is overexpressed in TAMs and ultimately promotes tumor growth. Importantly, we revealed that ACSL3 is lactylated at K679 in TAMs and that SIRT7-mediated ACSL3 K679 delactylation reduces ACSL3 activity, leading to increased ferroptosis and enhanced antitumor immunity. Meanwhile, the activation of ACSL3 activity promotes lipid droplet formation and ferroptosis resistance, sustaining the immunosuppressive phenotype of TAMs. These findings clarify a previously unappreciated mechanism by which tumor-derived lactate modulates TAM metabolism and function via non-histone lactylation.

Several studies have shown that inducing ferroptosis in TAMs can promote their transformation to an M1-like pro-inflammatory phenotype, while enhancing antigen presentation and increasing T cell infiltration and cytotoxicity [43–45]. In contrast, ferroptosis-resistant TAMs maintain an M2-like immunosuppressive phenotype that suppresses adaptive immunity and facilitates tumor progression [46]. These findings underscore ferroptotic regulation as a key determinant of macrophage-driven immune modulation within the TME [47]. Our study demonstrates that ACSL3 plays a pivotal role in maintaining ferroptosis resistance within TAMs. We found that ACSL3 undergoes lactylation at lysine 679 (K679), a modification that enhances its enzymatic activity and confers protection against lipid peroxidation–induced ferroptosis. This ferroptosis-resistant state enables TAMs to sustain their immunosuppressive phenotype, thereby inhibiting T cell cytotoxic activity and promoting CRC progression. Functionally, inhibition of ACSL3 in TAMs restores T-cell effector function and suppresses tumor cell proliferation. Collectively, these results identify ACSL3-mediated ferroptosis resistance as a crucial mechanism through which TAMs exert immune suppression and facilitate tumor development.

Taken together, our study identifies a SIRT7-ACSL3-K679 lactylation axis that links tumor-derived lactate metabolism, ferroptosis regulation, and TAM functional plasticity. Lactate promotes ACSL3 activity via K679 lactylation, resulting in lipid remodeling and ferroptosis resistance, which sustains an immunosuppressive TME. SIRT7-mediated delactylation counteracts this process, restoring ferroptotic sensitivity and enhancing T cell cytotoxicity. These findings provide a mechanistic framework for how metabolic cues in the TME modulate TAM function and tumor immunity, highlighting potential therapeutic targets for restoring anti-tumor immunity. Targeting the SIRT7-ACSL3 axis may offer dual therapeutic advantages: reprogramming tumor-associated macrophage (TAM) metabolism to make it sensitive to ferroptosis, while enhancing T-cell-mediated anti-tumor responses. Understanding these mechanisms may provide guidance for the development and utilization of combined strategies of metabolism-immune cross-communication in the treatment of CRC.

From a translational perspective, our findings suggest several potential therapeutic opportunities. Targeting ACSL3 lactylation or restoring SIRT7 activity may represent a promising strategy to reprogram TAMs from an immunosuppressive to an immunostimulatory state. Such approaches could be achieved through pharmacological modulation of lactate metabolism, direct inhibition of ACSL3 activity, or enhancement of SIRT7 function. Notably, given the increasing interest in ferroptosis-based therapies, combining ACSL3 targeting with ferroptosis inducers may further sensitize TAMs and tumor cells to lipid peroxidation, thereby amplifying anti-tumor efficacy. In addition, the differential expression patterns of ACSL3 and SIRT7 observed in TAMs suggest their potential utility as biomarkers. The combination of high ACSL3 expression and low SIRT7 expression may reflect an immunosuppressive microenvironment and disease progression, and could potentially be used to stratify patients who are more likely to benefit from metabolism-targeted or immunomodulatory therapies. Furthermore, given the growing application of immune checkpoint blockade in CRC, modulation of the SIRT7–ACSL3 axis may enhance therapeutic responsiveness by alleviating TAM-mediated immunosuppression and restoring T cell activity, providing a rationale for combination strategies.

Nevertheless, several limitations should be acknowledged. The clinical sample size in this study is relatively limited, and further validation in larger patient cohorts is required. In addition, while our data suggest a regulatory role of lactylation in ACSL3 function, the precise enzymatic mechanisms governing this modification remain to be fully elucidated. Future studies using clinically relevant in vivo models and pharmacological intervention will help determine the feasibility and potential therapeutic effects of cancer treatment targeting lactylation of ACSL3 in TAMs.

## Supplementary information

Western blots

Supplementary tables

S1

S2

S3

Supplementary Figure legends

## Data Availability

The data supporting the findings of this study are available within the article and its Supplementary Information files. Additional data are available from the corresponding author upon reasonable request.
