# Supplementary material for: SIRT7-mediated ACSL3 delactylation suppresses colorectal cancer progression by orchestrating tumor-associated macrophage ferroptosis and polarization
Source: Cell Death Dis. 2026 Jun 30;17(1):805. doi: 10.1038/s41419-026-09007-2 (PMC13582799; doi:10.1038/s41419-026-09007-2)
Supplement: Supplementary file 1 [file 41419_2026_9007_MOESM2_ESM.docx]

**Supplementary tables**

**Table S1** **Antibodies used in this study**

| Antibody | Source | Catalog.No | Dilution | Application |
| --- | --- | --- | --- | --- |
| Anti-ACSL3 | Abmart | TD9606M | 1:1000/1:50 | WB/IF |
| Anti-Lactyl Lysine | PTM | PTM-1401 | 1:1000 | WB |
| Anti-acetyl-lysine | Abmart | M30069S | 1:1000 | WB |
| Anti-Succinyl-lysine | PTM | PTM-0401 | 1:1000 | WB |
| Anti-Flag | Proteintech | 20543-1-AP | 1:5000 | WB |
| Anti-Actin | ABclonal | AC026 | 1:5000 | WB |
| Anti-SIRT7 | Proteintech | 12994-1-AP | 1:1000/1:50 | WB/IF |
| Anti-CD163 | CST | 93498S | 1:1000/1:100 | WB/IF |
| Anti-CD163 | Proteintech | 68218 | 1:200 | IF |
| Anti-HA | Proteintech | 51064-2-AP | 1:5000 | WB |
| Anti-Myc | Abmart | M20002 | 1:1000/1:200 | WB/IP |
| Anti-CD86 | Proteintech | 16623-1-AP | 1:1000 | WB |
| Anti-CD68 | santa cruz | sc-70761 | 1:1000 | WB，IF |
| Anti-CD8 | Proteintech | 85977-4-RR | 1:5000 | WB |
| Anti-CD8 | LBP | IP024 | 1:100 | IF |
| Anti-ki67 | STARTER | SOB2332 | 1:50 | IHC |
| Anti-ACSL4 | Proteintech | 66617 | 1:1000 | WB |
| Anti-SLC7A11 | Proteintech | 26864-1-AP | 1:1000 | WB |
| Anti-GPX4 | Proteintech | 30388-1-AP | 1:1000 | WB |

**Table S2 Primers for qPCR**

| ID | Sequence (5’-3’) |
| --- | --- |
| ACSL3-F | ATACGTTCGTCCCCTCGCAT |
| ACSL3-R | CCAGTCCTTCCCAACAACGA |
| Actin-F | AGTGTGACGTGGACATCCGCAAAG |
| Actin-R | ATCCACATCTGCTGGAAGGTGGAC |
| CD163-F | ACCTGCACTGGAATTAGCCC |
| CD163-R | CAGTGCCTGTTTTGTCACCAG |
| CD80-F | CTCTTGGTGCTGGCTGGTCTTT |
| CD80-R | GCCAGTAGATGCGAGTTTGTGC |
| CD206-F | GGGTTGCTATCACTCTCTATGC |
| CD206-R | TTTCTTGTCTGTTGCCGTAGTT |
| TNF-α-F | CTCTTCTGCCTGCTGCACTTTG |
| TNF-α-R | ATGGGCTACAGGCTTGTCACTC |
| IL10-F | AGGCAACCTGCCTAACATGC |
| IL10-R | GTTCTCAGCTTGGGGCATCA |
| INOS-F | GCTCTACACCTCCAATAATGTGACC |
| INOS-R | CTGCCGAGATTTGAGCCTCATG |
| IL1β-F | AACCTCTTCGAGGCACAAGG |
| IL1β-R | AGCCATCATTTCACTGGCGA |
| IL-6-F | AGACAGCCACTCACCTCTTCAG |
| IL-6-R | TTCTGCCAGTGCCTCTTTGCTG |
| Arg1-F | TCATCTGGGTGGATGCTCACAC |
| Arg1-R | GAGAATCCTGGCACATCGGGAA |
| TGF-β-F | GCAGCACGTGGAGCTGTA |
| TGF-β-R | CAGCCGGTTGCTGAGGTA |
| PRF1-F | TGACACAACACGGACAGAAC |
| PRF1-R | GCATCACAGGTATAACGGTAGG |
| GZMB-F | CAGACAGAACGCCAAGCATC |
| GZMB-R | TCGCACTCCTCAATCTCACC |
