# Supplementary material for: SIRT7-mediated ACSL3 delactylation suppresses colorectal cancer progression by orchestrating tumor-associated macrophage ferroptosis and polarization
Source: Cell Death Dis. 2026 Jun 30;17(1):805. doi: 10.1038/s41419-026-09007-2 (PMC13582799; doi:10.1038/s41419-026-09007-2)
Supplement: Supplementary file 2 [file 41419_2026_9007_MOESM6_ESM.docx]

**Supplementary Figure** **legends**

**Supplementary Figure 1. Flow cytometric validation of TAM phenotype under co-culture conditions**

(A) Single-cell RNA-seq data (GSE132257) from colorectal cancer tissues were analysed by UMAP to identify major cell populations. ACSL3 expression across cell subsets was visualized, with comparison between normal and tumor samples (two-sided Wilcoxon rank-sum test, P=0.0027).

(B,C) qRT–PCR analysis of M1 and M2 markers in M0, M1, and M2 macrophages.

(D,E) qRT–PCR analysis of M1 and M2 markers in macrophages co-cultured with NCM460 cells (control) or CRC cells (HCT116 or SW620, TAMs).

(F,G) Flow cytometry validation of surface ACSL3 expression in monocyte-derived macrophages under co-culture conditions. Representative plots of HLA-DR (APC-H, clone R675-H) and CD206 (PE-H, clone B572-H) confirm an ACSL3^high CD206^+ M2-like TAM phenotype.

Data are presented as mean ± SD (n=3). Statistical significance was determined using one-way ANOVA followed by Tukey’s multiple comparisons test for multiple group comparisons (B–E, G). Comparisons in panel G were performed relative to the M0 and NCM460 co-culture groups. ***P < 0.001, ****P < 0.0001.

**Supplementary Figure 2. ACSL3 deficiency suppresses tumor growth and enhances anti-tumor immunity**

(A) ACSL3 mRNA expression in TAMs after transfection with ACSL3-specific siRNAs (si-ACSL3#1 and si-ACSL3#2) or control siRNA (si-NC) and co-culture with Jurkat T cells, corresponding to Fig. 2D–E.

(B) Representative tumor images from a subcutaneous CRC model established by co-injection of MC38 cells and RAW264.7-derived macrophages (1:1).

(C-E) Tumor weight measurements, tumor growth curves showing tumor volume changes over time, and body weight monitoring of mice in the subcutaneous CRC model.

(F, G) Representative images and quantification of Ki-67-positive cells in tumor tissues. Scale bar, 250 μm.

(H) mIF staining of tumor tissues showing CD68^+^/CD163^+^ macrophage infiltration and CD8^+^ T-cell distribution. Scale bar, 50 μm.

Data are presented as mean ± SD (n=3). Statistical significance was determined using one-way ANOVA followed by Tukey’s multiple comparisons test for multiple group comparisons (A), a two-tailed unpaired Student’s t-test for comparisons between two groups (C-E, G). **P < 0.01, ***P < 0.001, ****P < 0.0001.

**Supplementary Figure 3. Extended validation of macrophage polarization markers in ACSL3-deficient TAMs**

(A–C) qRT–PCR analysis of additional M1 markers in ACSL3-deficient TAMs, corresponding to Fig. 5C, D.

(D–E) qRT–PCR analysis of additional M2 markers in ACSL3-deficient TAMs, corresponding to Fig. 5E, F.

Data are presented as mean ± SD (n=3). Statistical significance was determined using one-way ANOVA followed by Tukey’s multiple comparisons test for multiple group comparisons (A–E). ***P < 0.001, ****P < 0.0001.
